# Complexity and Health Coaching: Synergies in Nursing

**DOI:** 10.1155/2013/238620

**Published:** 2013-09-11

**Authors:** Gail J. Mitchell, Nadine Cross, Michelle Wilson, Shauna Biernacki, Winnie Wong, Behnam Adib, Danica Rush

**Affiliations:** ^1^School of Nursing, York-UHN Nursing Academy, York University, HNE 349, 4700 Keele Street, Toronto, ON, Canada M3J 1P3; ^2^University Health Network, York-UHN Nursing Academy, York University, Toronto, ON, Canada; ^3^Southlake Regional Health Centre, Newmarket, ON, Canada; ^4^University Health Network, Black Creek Community Health Centre, Toronto, ON, Canada; ^5^University Health Network, Dotsa Bitove Wellness Academy, Toronto, ON, Canada M4G 3E8

## Abstract

Health care professionals are increasingly aware that persons are complex and live in relation with other complex human communities and broader systems. Complex beings and systems are living and evolving in nonlinear ways through a process of mutual influence. Traditional standardized approaches in chronic disease management do not address these non-linear linkages and the meaning and changes that impact day-to-day life and caring for self and family. The RN health coach role described in this paper addresses the complexities and ambiguities for persons living with chronic illness in order to provide person-centered care and support that are unique and responsive to the context of persons' lives. Informed by complexity thinking and relational inquiry, the RN health coach is an emergent innovation of creative action with community and groups that support persons as they shape their health and patterns of living.

## 1. Introduction

What is the appeal of complexity thinking for nurses in practice and for nursing as a discipline? Possibly, it is the idea of *possibility *itself and the appeal of the reality of ambiguity and nonlinear change that typify nursing work. Nurses come from a place of complexity, a place of understanding lived experiences (suffering, hope, fear, and violence), a place of practices that lurk in the borderlands of other more defined disciplinary fields. Many nurses who work with persons in community have a deep and embodied understanding of the complexities, ambiguities, and possibilities of working toward some betterment with persons in the context of their complex lives. The purposes of this paper are to describe a relatively new nursing role, the registered nurse health coach (RNHC), and to explore the relationships among complexity thinking and the emergent practices of the RNHC in community settings.

## 2. The Registered Nurse Health Coach

The health coach is a new role in Canada, and the Ministry of Health and Long Term Care (MoHLTC) in Ontario may be the first ministry to designate specific resources to support nurses in the coaching role. The coaching role has been developed over the past decade in the United States, Australia, and the United Kingdom, and research about the effectiveness of the coaching role for helping persons with type 2 diabetes mellitus and other chronic illnesses is promising [1]. Prior to examining qualities and responsibilities of the health coach as conceptualized in our project, a brief view of health coaching is reviewed in a broader context.

Health professionals are increasingly acknowledging that persons living with chronic illness must be supported with a comprehensive and compassionate form of health care that goes far beyond simply providing education/information. The central qualities and role responsibilities of the health coach role found in a literature review are consistent with the role as it is developing in Canada. Several authors implementing the health coach in primary care, clinics, and community health settings specify that the health coach provides person-centred care within relationships that are based in respect and nonjudgmental attitudes and practices [2–5]. Coaches need to have excellent listening skills and to be able to offer support and care based on value-based principles, including a clear commitment to follow the lead of the person and his/her priorities and goals for change [3, 6–8]. Anderson and Funnell [3] suggest that the fundamental shift in an empowerment approach is where health workers “change from feeling responsible for patients to feeling responsible to patients” (page 155). Hayes and Kalmakis propose that coaching “focuses on the clients' feelings, desires, experiences, personal goals, discoveries, and learning” [9, page 557] and that a coaching role attends to who the person is and what they discover and see as possible.

The literature informs a foundation of deep respect and nonjudgmental practices with persons living unique situations, but this foundation is not yet buttressed with ontological or theoretical beliefs that further enable nurses to be creative agents in the nurse-person process, agents that can transcend the legacy of modernism and the rules of causality, prediction, and control. Complexity thinking and its affiliated basic tenets can provide the creativity and openness required for nonjudgmental compassion and respect in relationship.

## 3. Complexity Thinking

We have been deeply influenced by complexity thinking in our scholarship and work as educators and community nursing practitioners. It would be impossible to list all the authors who have helped us to forge a path of complexity in practice and education, but several must be cited [10–15]. We are in agreement with other nurse scholars such as Smith [16] and Gambino [17] that fundamental beliefs of complexity thinking fit conceptually with extant works of nurse theorists such as Rogers [18, 19], Newman [20, 21], and Parse [22, 23]. For instance, these three theorists, in particular, advanced ideas of unitary beings (greater than and different from the sum of parts), patterns of living/meaning and increasing complexity, mutual process, and nonlinearity [24]. Complexity thinking too embraces these ideas and expands the discourse of living beings/systems across disciplinary silos and turns our attention to emergence/learning/change, to inclusivity, to *both and *thinking, and to the interplay of discourse and relationships that inform our human work and human community.

Other nurses are embracing complexity thinking and forging insights that focus on the synergies between complexity and nursing. For instance, Lindberg et al.'s [25] compilation of writings from nurses and others highlights how readily complexity ideas fit with nursing practice, theory, research, and leadership. The articles relate the ease of pattern identification and relationships, fundamental ideas for the health coach role. Further, chapters in the Davidson [26] text—*Complexity and Nursing*—invite readers to consider complexity and possibility and how these ideas are reflected within the breadth of nursing. Aspects of complexity thinking, specific to relationships assisted Colón-Emeric et al. to understand how differing patterns of relationship and communication enable the flow of information, diversity, and innovative care [27]. For some time leaders in health care organizations have been exploring the central tenets of complexity thinking and how they inform leadership and decision making [28, 29]. Such literature has guided change in organizational viewpoints—from one of health care as a machine with isolated parts, to considering organizations as complex systems with parts always in relationship. The web site Plexus offers a broad array of resources and connections (http://www.plexus.org).

From our perspectives, complexity thinking is informing the practices of the RNHC as an emergent, relational process. Relational inquiry has been articulated, as a foundation for nursing practice by Doane and Varcoe [30], and we build on their insights in the relationships we have established (in practice and research) with persons living with diabetes. In particular the RNHC practices described here have moved away from the medical gaze of assessing and evaluating persons toward a gaze of relating, reflecting, and acting with clients. The RNHCs have developed comfort in the ambiguous borderlands where people articulate complex and pressing needs in a system that is not yet prepared to accept the reality that health care delivery is a political affair with weak, if not failing, connections to the realities of poverty, accessibility, violence, inequality, mobility, and need. Complexity thinking helps prepare the RNHCs for dwelling in the uncertainty of nonlinear change and transformation. Complexity helps the RNHCs to *resist *the dominant discourse that places accountability for illnesses like diabetes almost exclusively on “unmotivated” individuals who are seen as needing to change their lifestyles and day-to-day choices. Complexity helps the RNHCs to stay open during turbulent times and to hold the belief that we are all part of a much larger living system that is also emergent and self-creating through relationships. And complexity informed pedagogy helps nurses to understand that we cannot educate other people by giving information; understanding and learning are linked with an essential process of conversation and insight [31], imagination and recursion [11].

Based on these core insights, the educators and researchers involved in the development of this new RNHC role in Southern Ontario, Canada, developed value-based competencies to help guide their relational processes with persons living with diabetes. The competences the group developed are as follows.

*The RNHC:*

 creates and sustains a relational inquiry that promotes health through pattern recognition and change,
 integrates concepts from complexity science to help people stretch and change their understandings and views,
 uses tools/activities to help people explore their readiness to change and their patterns of self- and family care,
 assists people to explore healthy routines by enabling self-knowledge and self-care activities in light of issues of social justice and accessibility,
 works with families and communities to illuminate both assets and barriers for self-care and wellbeing,
 establishes partnerships with community organizations to enable health promoting activities,
 provides leadership to health professionals and organizations to extend health promotion and health coaching,
 mentors students and other professionals in health coaching competencies,
 advocates for structural changes that enable health promotion for groups and communities,
 participates in research activities and contributes to knowledge creation and dissemination.

The RNHCs continue with teaching/learning workshops at York University pertinent to the health coach role. They are also engaged in curriculum development for health- promoting projects. For example, the RNHCs partnered with community pharmacists to create *Café Diabetica*, an arts-based program of personal discovery and engagement for persons living with diabetes. The partnership and pilot of the community engagement project were very well-received, and the team is planning a larger study. 

## 4. Context of the RNHC

The four RNHCs in our project were hired in the summer of 2011. Two RNHCs were positioned with the Outreach Chronic Disease Program at a Regional Health Centre (RHC). This 300 bed community hospital is located in York Region on the northern flank of a large metropolitan area. The Region is comprised of nine municipalities both suburban and rural. The second setting is a Community Health Centre (CHC) located in the northwest region of the same metropolitan city.

The CHC has a wide urban catchment area that services approximately 200,000 persons. This community is known for its diversity and rich cultural patterns. The health care emphasis at both sites is to address the prevalence of chronic health conditions and the low utilization rate of health services by reducing health inequities through a health promotion approach that attends to the culturally and socially diverse communities. Each site has an existing diabetic educational service staffed with educators, social workers, physicians, and dieticians. The RNHCs partner with persons who are experiencing type 2 diabetes and who live in the regional community

## 5. Referral Systems, Work Loads, and Patterns of Support

The RN health coaches (RNHCs) have a work load of approximately *75+ active *persons/families/groups for each site, and each pair responds to about 90 phone calls per month. The health coaches visit persons in apartment buildings, community centres, libraries, pharmacies, and persons' homes, as needed in order to create and sustain relationships. As well, the RNHCs provide presentations on the role of the health coach to community and professional groups such as diabetes education teams, family practice units, geriatric outreach teams, and pharmacies. Sources of referral received by the RNHCs range from the person him/herself to health professionals and staff at urgent care clinics. Reasons for referral included issues relating to complex personal situations, frequent episodes of diabetic ketoacidosis, financial and food insecurity, and solitude.

## 6. Preliminary Impressions of Changes with RNHC Role

Preliminary evaluation of the RNHC nurses is promising. Professionals and persons/families/groups are very interested in the role and are referring and working with the health coaches. The nurses report satisfaction with the role and the education/support being provided. We have received reports of significant change in patterns of self-care for some persons working with the RNHCs. The impact of the RNHC role for patients/families will be systematically evaluated through quality management initiatives (surveys, telephone calls) and more rigorously in funded research projects.

In general, the health coach role is receiving increased interest in the United States and Canada [32]. The role has made significant improvements in models of chronic disease care, health promotion, and decreased visit to emergency services in the United States. The four RNHCs in York Region are making important contributions to existing health teams and patient outcomes. Plans are to grow the role as opportunities present and to continue evaluation over next year.

## 7. A Composite Story

Coming to understand the carefully nuanced patterns of relating between the RNHC and persons living with diabetes is a multifaceted experience as such we offer the reader a story. This story is a composite of the many experiences emergent from the RNHC practice. A composite narrative [33] offers an embodied understanding that authenticates the truths of lived experience and chronicles the richness of multiple voices and the importance of interpretation.

Referrals for the registered nurse health coach (RNHC) program are similar to referrals to any other outpatient program. Health professionals, who have concerns about a patient with diabetes and the person's ability to self-manage, will make a referral to the RNHC program.

The referrals provide an opportunity for persons to collaborate one on one with the health coach in order to explore the issues and meanings of health in the context of their lives. The RNHC offers persons the opportunity and space to engage in open dialogue, in a safe environment that is free of judgment. The influence of judgment in health care has not been fully explored, and in our experience it is as important as other determinants of health. Indeed, we believe that judgment, blame, and stigma form a cluster that constitutes a significant barrier to health care, but we digress.

The RNHC creates a relationship where persons can explore their own issues and interests, their own views and concerns, fears, and hopes. Complexity thinking helps the nurses to understand that all change emerges from the past and that persons self-organize or self-create their understandings and actions according to what makes sense in their own lives. The RNHC lived the intent to understand and to follow the person's lead as s/he explores the deeper patterns of life and lived relationality. We now describe a situation with a man named Joe, who was referred to an RNHC from a member of a diabetes education team.

Joe recently begun insulin therapy and had been experiencing frequent hypoglycemic episodes. During my initial home visit, he shared some of his 30-year journey of living with diabetes. Having experienced multiple hospitalizations in the past, he explained that his most recent episode was due to a mixup between short acting and long acting insulins. Although he had accessed self-management courses in the past and was working closely with the diabetes education team in order to monitor and manage his diabetes, it was evident that other issues in his life had taken precedence and diabetes was not at the forefront of his concerns. Joe, like many others, had been provided an abundance of diabetes-related information. He had contact with diabetes education teams, various nursing and dietician supports, group wellness programs, government funded self-management programs, and community-based support groups. But, Joe taught me that people's lives are so much more complex than just the disease.

Most health professionals know, even in their own lives, that information does not change behavior. Changing life habits are extremely difficult. We ourselves, or our loved ones, struggle with the revolving diet door, the promises to start or stop tomorrow, and the resolutions and commitments that we know we want but do not achieve. What is this human phenomenon? How can we better understand it so that we can begin to really help people to make changes that improve their health and wellbeing? We know the things we do and how we feel are related to our experiences in life, our interactions, our upbringing, and our overall journey in life. These influences are intertwined with the world we live in and how we react to it. People living with diabetes or any health condition bring their experiences and feelings that can only be surfaced in the presence of unconditional regard and compassion.

Joe was not only living with an illness that affects him physically, but he was trying to find a balance between the emotional burdens of diabetes and everything else in his life. He had been living with depression for a number of years and realized that life events and diabetes both affect his mood. He identified a pattern that his depression affects how he deals with and handles those life situations, such as his divorce and recent loss of a family member. Such a pattern even affects his blood sugar levels despite his efforts with daily exercise and healthy eating. The implications of the patterns made him frustrated and wanting to indulge in food that he knows will also raise his blood sugar level; “they are already so high…so why the hell not?” he asks me. Good question I think, reflecting on how I would react if the roles were reversed, probably the same, even though I am a health professional that is aware of the outcomes if optimal blood sugar control is not maintained.

During our conversation, I proposed a question asking Joe to think about an area where he felt he could see change that would lead to improving his current situation. His initial reaction was “you are the expert, I am coming to you to tell me what to do and you're asking me?” In response, I offered that he is the expert in his own life and his expertise is seen in how he has lived with diabetes over the past 30 years. I explained that as a nurse I could provide an array of information and several resources for what to do and how to do it; however, Joe had acknowledged that he has the information. As a way to begin a relational partnership with Joe, I wanted to accompany him through the process of exploring what changes he felt he could make and how he thought he could approach them; it seemed simple enough. I went on to say, “you are the most knowledgeable about yourself, your current situation and what has worked for you in past situations, so yes I am asking for your direction, I can tell you what you need to do, but would you do it?” He looked at me, and I could see him smile. In that moment we connected and knew that only he could decide how and what would work for him in his life. He had the answers; maybe I could help him find them.

Getting to know persons and understanding the patterns of their lives through open dialogue and trust and without judgment are fundamental to the RNHC role. Understanding that change comes from within and occurs only when the individual is at a point where they are truly ready and that big changes often require a process of starts and stops. The success of change is related to how the person is able to sustain it and how environmental influences constrain or enable the change; change is fluid and can regress or flow forward. Change is as much a community affair as an individual pursuit when it comes to health, because poverty, mobility, and accessibility are shared responsibilities of communities.

At this point, Joe said he was not ready to make changes due to the overwhelming issues he was currently experiencing. He began to tell me about an experience he had a day prior where he felt he had no control over the drop in blood sugar level, and he attributed it to having started insulin. Joe said, “when you are on insulin the highs and lows are just something you have to live with.” We explored his thoughts about the cause of his low blood sugars, and he indicated that it was due to “taking too much insulin or not eating enough.” We explored his feelings about how he decides about the amount of insulin he takes and food he eats. Joe lives with limited financial resources for accessing food, and the physician directed him to use a sliding scale insulin dose. Through our dialogue, Joe began to realize that he had the knowledge about which foods affect his bloods sugars and in which way. He then looked at me in silence, and at that moment he seemed to have new insight about his diabetes; the insight changed his understanding, perhaps, or shifted the angle he had on the control issue.

As I listened to Joe speak and tease apart his thoughts on his blood sugar control, he spoke about feelings of fear and shame that surfaced when he attended appointments with healthcare providers. He felt like he was “wasting their time” which led him to avoid appointments altogether. Inquiring further, he stated that he always tries his best; however, life events seem to get in the way, increasing his stress level and affecting his blood sugar numbers. He explained that during some appointments there had been some serious communication expressed to him about his inability to improve his blood sugars and self-management routines. At one point, Joe was instructed to decrease his stress level, period. This seems like such an absurd directive, and the instruction itself contributes to the person's stress. Joe is still struggling to reduce the stress in his life, but he does not know how. I wondered how someone could reduce the stress of a divorce or the death of a family member. The idea of community comes to mind again here, but community is not an important concept in modern health care. Sadly, community is thought of as a place, not a relation.

Over the next two months we discussed some of Joe's life stresses, which included housing, access to healthy foods, financial struggles, and mobility. I assisted him with accessing his local food bank, and with the help of the Community Care Access Centre (CCAC) he obtained a walker and other home safety equipment. As our relationship grew and we continued to develop trust, Joe continued to slowly reveal his deeper struggles of living with diabetes. He spoke about having fear of diabetes when he was a child, watching his grandfather die of the disease, and feeling threatened by family members of “catching” diabetes if he ate too much sugar. Many of our conversations cycled and intertwined with multiple aspects of his life, past, present, and future. By paying close attention to his stories, I was able to notice that he was reflecting more and more on how he felt about diabetes that day. In addressing some issues within his situation of resources and accessibility, things that have hindered Joe's self-care in the past, diabetes was becoming more of a priority for him. He recognized the need for attention to the diabetes portion of his life but felt overwhelmed in trying to deal with the other aspects of life that often took priority.

During our subsequent visits, Joe and I explored the idea of control with his fluctuating blood sugars, and he started using a calendar to track only his low blood sugars along with the reasons why he felt they had occurred. I followed up with him one month later, and his calendar was blank. He explained to me that he was better able to manage the sugars with his current practice of home monitoring and adjustment of dose. We reviewed his new process of care, and he said that he felt the calendar was helping him to be more accountable for his sugar levels. Joe started attending his appointments more regularly and was very excited to share with me that he and the diabetes education team felt that he was doing so well that he would be able to extend his visits to every six months. Joe and I keep in close contact and are able to discuss any issues or concerns he may have with his self-care plan.

As a RNHC, my role is not to change persons living with diabetes. Instead, I hope to better understand their struggles as they see and experience them and provide them with the support to navigate through the challenges they face with self-care while connecting with community resources. Persons commit to exploring change when they feel they are ready and have the support and resources to do so. Joe's situation helps show how health coaching that is informed by relational principles and complexity thinking can make a difference. It is very satisfying work when you can let go of thinking that health professionals have control of others when, in truth, we can only control our intent with others. We have come to understand that the consequences of relationships emerge through the engagement.

## 8. Our Emergent Learning

As a community of nurses committed to health coaching, especially for persons living with chronic illness and change, we have new embodied understanding about how complex systems are learning systems. We have seen that it is through the intent to be in relation with diversity and perturbations within the porous borders that learning emerges. Our learning with implementation of the RNHC role is to understand it as robust, ever evolving, and constantly shifting with the learning systems coconstituting the nested realities of health and society. The form, qualities, responses, relationships, and patterns of the existing system have changed with the presence of the RNHCs and the system has learned. Change is critical, as our existing health care system is not as effective as it could be. Specific lessons we would like to share here include the following.

1. Persons, like organizations, are complex systems who have nested histories and embedded experiences that shape their emerging patterns, feelings, and actions. Turbulence and calm coexist in living systems. The RNHCs in partnership with the person also form a complex system within the larger health care system, and the health care system is part of a political and regulatory system, and all interrelate in many different ways. Persons are affected—for better or worse—through the relationships and politics of the communities they engage.
2. Complex systems are living, self-organizing, and evolving unities where patterns, feelings, and relationships become generative and informative. As such the ideas of networked, nested structures, dissipative hierarchy, disequilibrium, and perturbations (put out of kilter) coexist. We have experienced these intersections of disequilibrium/perturbations, as the RNHCs moved within existing structures and with each new RNHC-person relationship. We have learned that places of ambiguity and uncertainty are also places of discomfort and possibility.
3. Complex systems have porous, blurry borders as we are always connecting and disconnecting with others in layered surroundings. The RNHCs experienced the ambiguity of working out a new role in the presence of challenge and suspicion from colleagues and persons in community. Through their intent to understand and build relationships with persons living with diabetes, they were able to contribute new perspectives—some that clarified and others that disrupted assumptions and habits of care.
4. The recursions/iterations and nonlinear dynamics of introducing the RNHC role were lived out and experienced in networks developed with communities, hospitals, families, and health professionals. Like all complex systems the changes introduced by the RNHC cannot be known through simplicity of linear models. We will need to study the role from multiple perspectives over time to gain some insights about the impact of the RNHC.
5. Understanding patterns of relating is fundamental to the RNHC role. The patterns are the intertwining of events, ideas, and persons in relationship that create a complex unity. The unity of complexity points out difference and diversity as desired features of growth and learning. Complexity teaches us that a system must have diversity in perspectives for persons to thrive, and it is in relationships that we come to understand. The RNHCs developed their understanding of the patterns and relationships for persons living with diabetes.

We all need to keep learning and changing in unexpected ways through quality relationships. The RNHC role is one way to provide innovation and creative action in supporting persons to shape their health and self-care patterns.
